# Recombinant-attenuated *Salmonella enterica* serovar Choleraesuis vector expressing the PlpE protein of *Pasteurella multocida* protects mice from lethal challenge

**DOI:** 10.1186/s12917-023-03679-0

**Published:** 2023-08-19

**Authors:** Guodong Zhou, Jiashuo Tian, Yichen Tian, Qifeng Ma, Quan Li, Shifeng Wang, Huoying Shi

**Affiliations:** 1 College of Veterinary Medicine, Yangzhou University, Yangzhou, 225009 Jiangsu People’s Republic of China; 2 Jiangsu Co-innovation Center for the Prevention and Control of Important Animal Infectious Diseases and Zoonoses, Yangzhou, 225009 China; 3 Department of Infectious Diseases and Immunology, College of Veterinary Medicine, University of Florida, Gainesville, FL 32611-0880 USA; 4 Joint International Research Laboratory of Agriculture and Agri-Product Safety, Yangzhou University (JIRLAAPS), Yangzhou, China

**Keywords:** Recombinant attenuated *Salmonella* vaccine, *P*. *multocida*, PlpE, Vaccine candidate, Immune responses

## Abstract

**Background:**

Bacterial surface proteins play key roles in pathogenicity and often contribute to microbial adhesion and invasion. *Pasteurella* lipoprotein E (PlpE), a *Pasteurella multocida* (*P. multocida*) surface protein, has recently been identified as a potential vaccine candidate. Live attenuated *Salmonella* strains have a number of potential advantages as vaccine vectors, including immunization with live vector can mimic natural infections by organisms, lead to the induction of mucosal, humoral, and cellular immune responses. In this study, a previously constructed recombinant attenuated *Salmonella* Choleraesuis (*S*. Choleraesuis) vector rSC0016 was used to synthesize and secrete the surface protein PlpE of *P*. *multocida* to form the vaccine candidate rSC0016(pS-PlpE). Subsequently, the immunogenicity of *S*. Choleraesuis rSC0016(pS-PlpE) as an oral vaccine to induce protective immunity against *P*. *multocida* in mice was evaluated.

**Results:**

After immunization, the recombinant attenuated *S.* Choleraesuis vector can efficiently delivered *P. multocida* PlpE protein in vivo and induced a specific immune response against this heterologous antigen in mice. In addition, compared with the inactivated vaccine, empty vector (rSC0016(pYA3493)) and PBS immunized groups, the rSC0016(pS-PlpE) vaccine candidate group induced higher antigen-specific mucosal, humoral and mixed Th1/Th2 cellular immune responses. After intraperitoneal challenge, the rSC0016(pS-PlpE) immunized group had a markedly enhanced survival rate (80%), a better protection efficiency than 60% of the inactivated vaccine group, and significantly reduced tissue damage.

**Conclusions:**

In conclusion, our study found that the rSC0016(pS-PlpE) vaccine candidate provided good protection against challenge with wild-type *P*. *multocida* serotype A in a mouse infection model, and may potentially be considered for use as a universal vaccine against multiple serotypes of *P*. *multocida* in livestock, including pigs.

**Supplementary Information:**

The online version contains supplementary material available at 10.1186/s12917-023-03679-0.

## Background

*Pasteurella multocida* (*P*. *multocida*) is an important opportunistic bacteria that causes a wide range of infections including fowl cholera, bovine haemorrhagic septicaemia, rabbit pasteurellosis, swine atrophic rhinitis [1]. At the same time, it is also an important zoonotic pathogen that can cause serious abscess formation, meningitis, and leading to significantly harmful impacts on human health [1, 2]. Based on its capsular composition, the *P*. *multocida* is divided into A, B, D, E and F capsular serogroups and further classified into 16 serotypes (1–16) based on distinct lipopolysaccharide structures [3, 4]. Progressive atrophic rhinitis (PAR) and pneumonic pasteurellosis (PN) in pigs are the main diseases caused by toxigenic and nontoxigenic strains of types A and D *P*. *multocida* [1, 5]. As an important pathogen of pigs, *P*. *multocida* can coinfect with other pathogens including porcine reproductive and respiratory syndrome, porcine circovirus and *Mycoplasma hyopneumoniae* [6, 7]. This coinfection may cause immunosuppression to reduce the host’s immunity and resistance to other diseases.

Antibiotics therapy is usually used for the treatment of *P*. *multocida* infection [1, 8]. However, under intensive feeding conditions, the extensive use and misuse of antibiotics has led to the increasing resistance of *P*. *multocida*, which eventually has led to treatment failure [8]. Previous outcome studies also indicate that antibiotic resistant bacteria may also increases the potential risk of gene transfer to non-resistant bacteria and therefore may pose a risk to human health [9]. Vaccination is currently the most effective measure to prevent of *P*. *multocida* infection and disease [10]. Conventional inactivated *P*. *multocida* vaccine has been widely used and has played a crucial role in control and prevention of diseases caused by *P*. *multocida* globally [11]. However, traditional inactivated vaccines have many shortcomings, including thermal instability, short duration of immunization, weakly immunogenic, poor cross-protection, and inflammation at the injection site [10, 11]. The application of live attenuated vaccines, although showed a better protective efficacy, carries the risk of systemic infections after immunization as a result of potential reversion to virulence [10]. At present, inactivated vaccine (C44-1 strain) and live attenuated vaccine (EO630 strain) are two traditional vaccines commonly used in China. Immune responses and the protection in a mouse model were evaluated and found that both live attenuated vaccines (71%) and inactivated vaccines (51%) could not provide complete protection against homologous serotypes and did not protect against heterologous serotypes [11]. To address the above research questions, researchers have evaluated new types of vaccine such as subunit vaccines and DNA vaccines [12–15]. However, the correct folding, post-translational modification of proteins and harsh storage conditions still limit the development of related vaccines.

*P*. *multocida* exhibits its pathogenicity via numerous virulence factors, including adhesins, toxins, and outer membrane proteins [2, 4]. At the same time, these proteins are an ideal target antigen for a *P*. *multocida* protective vaccine. The PlpE protein is a surface protein and is considered as one of the major antigens among *P*. *multocida* for vaccine development [16]. After immunization, recombinant protein PlpE provided 80–100% protection in mice and induced cross-serotype protective immunity [13, 14, 17]. It also protected chickens against fowl cholera challenge [17]. Importantly, this protein was present in all *P*. *multocida* serotypes with the sequence identity over 90% [17]. These findings suggested that PlpE is an ideal target antigen for the development of anti-*P*. *multocida* vaccines. Of note, the recombinant protein-based vaccines often require different types of adjuvants to elicit a high levels of protective immune [18]. Live attenuated *Salmonella*, as an immune modulator and vaccine adjuvant, induces strong mucosal antibody and cell-mediated immune responses, has been successfully developed and used as a vaccine vector, and is an attractive platform for delivering various heterologous antigens to the immune system [19]. Over the years, achieving a balance between attenuation and immunogenicity has proved remarkably difficult: some live attenuated *Salmonella* vaccine vectors have been insufficiently attenuated, whereas others were over-attenuated but insufficiently immunogenic [20]. In order to address these questions, Roy Curtiss and colleagues have described a variety of novel strategies for the construction of attenuated vector, including regulated delayed attenuation, regulated delayed antigen synthesis, and regulated delayed lysis (for balance between safety and immunogenicity) [21–23]. At the same time, by constructing a balanced lethal system using complementation of the *asd* (aspartate semialdehyde dehydrogenase) gene *in trans*, to ensure the stability of the plasmid and the synthesis of heterologous antigens [22]. The *Salmonella* vector modified by the above system has been verified to deliver antigen proteins of different pathogens and to strengthen the antigen-specific antibody and T-cell immune responses, which can provide the host with more effective and durable immune protection [24–27]. These studies fully demonstrate that the attenuated *Salmonella* vector vaccine has great potential as a novel vaccine for protection against heterologous pathogens.

In previous research, our laboratory constructed a recombinant attenuated *S.* Choleraesuis vector containing the regulated delayed attenuation system and regulated delayed exogenous synthesis system, which we have named rSC0016 [27]. At the same time, its *sopB* gene was also knocked out to reduce the host intestinal inflammatory response induced by the *Salmonella* vector itself [26]. In this study, we constructed a recombinant plasmid based on the recombinant attenuated *S*. Choleraesuis vector expressing heterologous antigen PlpE, and assessed its immunogenicity and protective effect in mice. The results serve as a solid foundation for further development of an efficient and inexpensive universal vaccine against *P*. *multocida* disease for livestock, including pigs.

## Results

### Identification of ***P***. ***multocida*** serotype A and LD_50_ results

**Fig. 1:**
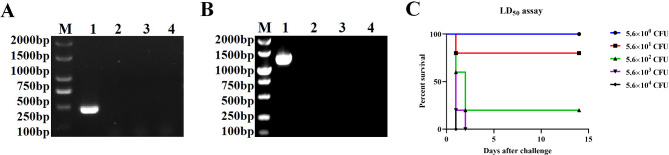
Identification results of strain species and serotypes. (**A**) *P. multocida* species-specific polymerase chain reaction for the detection of *KMT* gene (~ 460 bp). M: DL2000 DNA marker; lane 1: DNA of *P. multocida* serotype A field strain; lane 2: no DNA (negative control); lane 3: C78-3 DNA (*S. Choleraesuis* wild-type control); lane 4: DH5α DNA (*E*. *coli* control). (**B**) Amplification of *hyaD*-*hyaC* gene (~ 1044 bp) specific to *P. multocida* serotype A. M: DL2000 DNA marker; lane 1: DNA of *P. multocida* field strain; lane 2: no DNA (negative control); lane 3: C78-3 DNA (*S. Choleraesuis* wild-type control); lane 4: DH5α DNA (*E. coli* control). (**C**) Survival rate of mice (n = 5) challenged with different doses of *P. multocida* serotype A

Amplification with species-specific PCR successfully confirmed the isolate as *P*. *multocida* by amplification of DNA fragments (*KMT*; ~ 460 bp) (Fig. 1A). *P*. *multocida* capsular typing proven by the presence of the *hyaD*-*hyaC* gene of serotypes-A specific and amplified product, showed a fragment size of ~ 1044 bp (Fig. 1B). These results demonstrated that the isolate from diseased pigs was confirmed as *P. multocida* serotype A. Both primers failed to amplify the expected size bands in *Salmonella* and *E. coli*, indicating the specificity of the primers. To determine whether the *P*. *multocida* field strain was lethal to BALB/c mice and to measure the LD_50_ of the strain, six-week-old mice were intraperitoneally (i.p.) injected with serial dilutions of inocula of the bacterial strain. The test results indicated the LD_50_ of *P*. *multocida* serotype A field strain in BALB/c mice was 1.6 × 10^2^ CFU by i.p. administration (Fig. 1C). Our results showed that the *P*. *multocida* serotype A field strain was classified as highly virulent.

### Construction of rSC0016(pS-PlpE) and preparation of the inactivated ***P***. ***multocida*** serotype A vaccine and phenotypic analyses

**Fig. 2:**
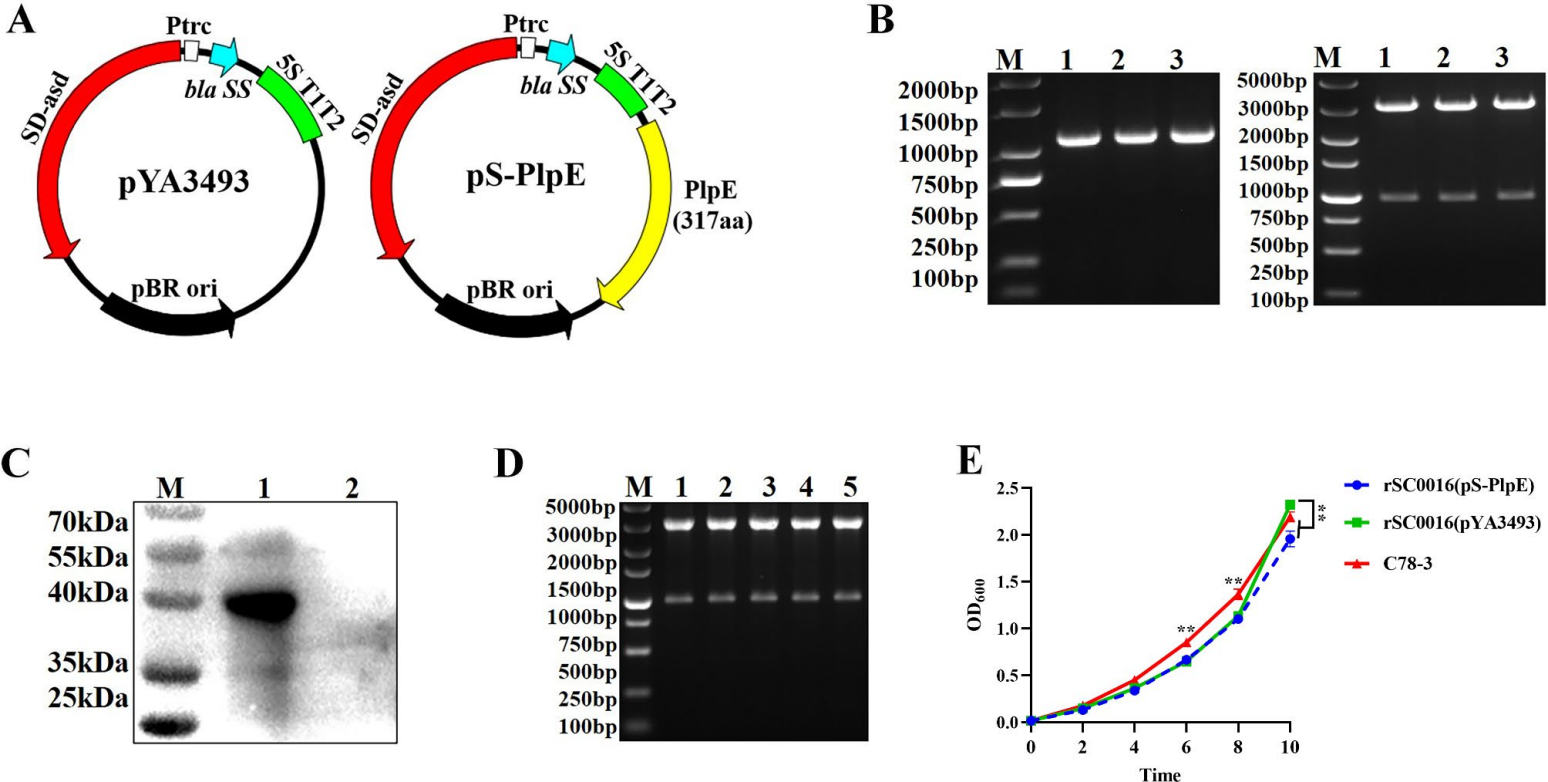
Plasmid maps and construction results, phenotypic characteristics of vaccine candidate strains. (**A**) Plasmid maps of pYA3493 and pS-PlpE. (**B**) Identification of recombinant plasmid pS-PlpE by PCR and restriction enzyme digestion. M: DL2000 or DL5000 DNA marker; Lane 1, 2, 3: *Eco*R I and *Sal* I digested pS-PlpE (different clones). (**C**) The expression of PlpE in rSC0016 was analyzed by WB. Lane 1: purified PlpE protein; lane 2: BL21(pET-28a). (**D**) Plasmid stability of pS-PlpE during passage. M: DL5000 DNA marker; Lane 1: 10 passages; lane 2: 20 passages; lane 3: 30 passages; lane 4: 40 passages; lane 5: 50 passages. (**E**) Growth curves of the rSC0016(pS-PlpE), rSC0016(pYA3493), C78-3 strains in the LB medium

The maps of the plasmids employed in the present study are presented in Fig. 2A. Successful construction of the plasmid pS-PlpE was confirmed by PCR amplification, *Eco*R I and *Sal* I digestion (Fig. 2B). The plasmid pS-PlpE that was constructed was further verified by DNA sequencing (data not shown), and the protein expression in the candidate vaccine strain was verified using polyclonal antiserum prepared as described in Methods. The expression of PlpE in the attenuated *S*. Choleraesuis vector is the basis for inducing protective immunity against *P. multocida* infections using the mouse infection model.

As shown in Fig. 2C, a band of 40 kDa of the expected size of the recombinant protein could be detected in rSC0016(pS-PlpE), but not in rSC0016(pYA3493), the empty vector strain. To determine the stability of our plasmids upon serial passage over time, individual transformants were picked into LB medium and transferred into fresh medium every 12 h for 50 passages. At passages 10, 20, 30, 40 and 50, the plasmids were extracted and identified by double enzyme digestion (Fig. 2D). Our results demonstrated that the use of Asd as a plasmid retention system (balanced lethal system) was adequate to enable plasmid retention in the Asd^−^ strain, rSC0016. The growth curve of the rSC0016(pS-PlpE) strain was also determined parallel to the rSC0016(pYA3493) strain and the wild-type *S*. Choleraesuis strain, C78-3. As revealed in Fig. 2E, the growth rate of wild-type strain C78-3 was significantly higher between 6 and 8 h when compared to other strains. It appears that rSC0016(pS-PlpE) grew somewhat slower than the rSC0016(pYA3493) between 6 and 8 h. However, the growth capacity was significantly higher in the rSC0016(pYA3493) compared to the rSC0016(pS-PlpE) strain at 10 h, the last time point. This indicates that foreign antigen expression may influence the growth capacity of the rSC0016 vector. After the inactivated vaccine was prepared from the wild type *P*. *multocida* serotype A strain, it was coated onto TSA solid medium containing 5% fetal bovine serum, and the results showed that no colonies grew (data not shown). The lack of colony growth on the solid media confirmed that the inactivation was sufficient, and the *P. multocida* inactivated vaccine was successfully prepared.

### High levels of antigen-specific antibodies were elicited by immunization with rSC0016(pS-PlpE)

**Fig. 3:**
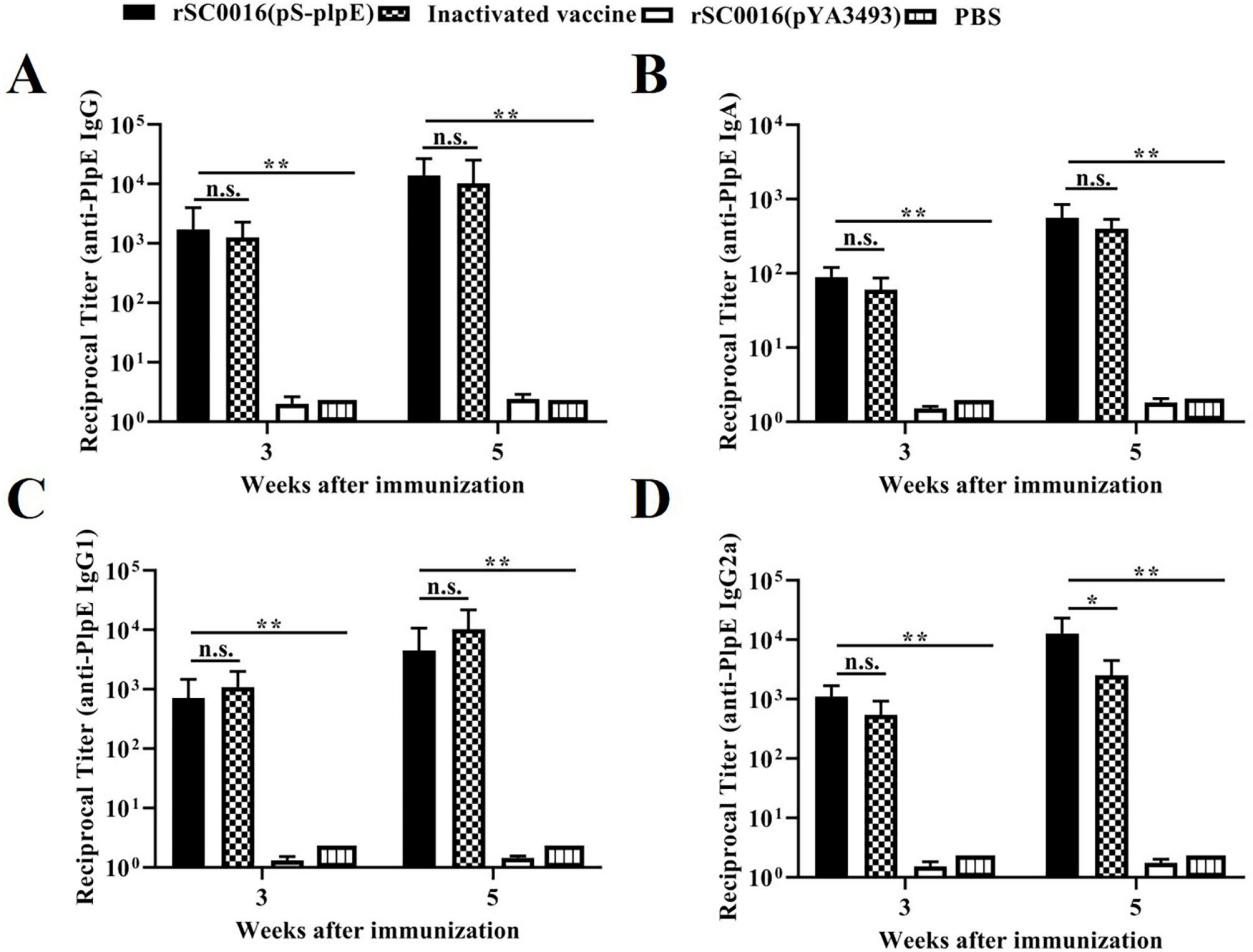
Detection of antibody titer in the immunized mice (n = 30). (**A**) PlpE-specific IgG antibody titer in serum determined by iELISA. (**B**) IgA titers detected by iELISA in vaginal lavage fluids. Specific anti-PlpE serum IgG1 (**C**) and IgG2a (**D**) titers. The results are expressed as the mean ± SD. Degrees of significance are indicated as follows: *P < 0.05; **P < 0.01; n.s., nonsignificant (*P* ≥ 0.05)

Specific antibody responses elicited in different immune groups were measured by titrating the serum and vaginal lavages of vaccinated and control mice against the PlpE protein. Compared with the PBS group or the rSC0016(pYA3493) empty vector group, mice orally immunized with rSC0016(pS-PlpE) and inoculated subcutaneously with the inactivated vaccine induced higher serum IgG titers against PlpE (Fig. 3A). Also, these levels were significantly higher in serum of mice after the booster immunization compared to those immunized with PBS or empty vector group. However, there was no significant difference between the rSC0016(pS-PlpE) and inactivated vaccine immunized groups. sIgA antibodies plays a critical role in mucosal immunity against *P*. *multocida* infection, so levels of sIgA were analyzed in vaginal lavage fluid collected from mice. As shown in Fig. 3B, both attenuated rSC0016 (pS-PlpE) and inactivated vaccine immunizations induced significantly higher levels of sIgA titers in mice than in the PBS and empty vector control groups. Although the mucosal immune response induced by rSC0016(pS-PlpE) strain was marginally higher than that of the inactivated vaccine immunization group, there was no significant difference. These data suggest that the rSC0016 (pS-PlpE) strain elicited significant specific humoral and mucosal immune responses, and the levels were comparable to those of the inactivated vaccines.

In mice, IgG2a is associated with the Th1-like immune response, while IgG1 is related to the Th2-like immune response. To further assess the types of immune responses in immunized mice, we examined IgG1 and IgG2a antibody subclass levels in the different groups (Fig. 3C, D). The results showed that mice immunized with rSC0016(pS-PlpE) and the inactivated vaccine induced increased levels of IgG1 and IgG2a, which were significantly higher than those immunized with PBS and empty vector. The inactivated vaccine induced higher, but not significant, IgG1 levels than rSC0016(pS-PlpE), while rSC0016(pS-PlpE) induced significantly higher IgG2a levels compared to the inactivated vaccinated group. The above results indicate that significant Th1- and Th2-type immune responses were induced in both the rSC0016 (pS-PlpE) and inactivated vaccine groups compared to the PBS and empty vector control groups.

### rSC0016(pS-PlpE) induced Th-1 and Th-2 responses

**Fig. 4:**
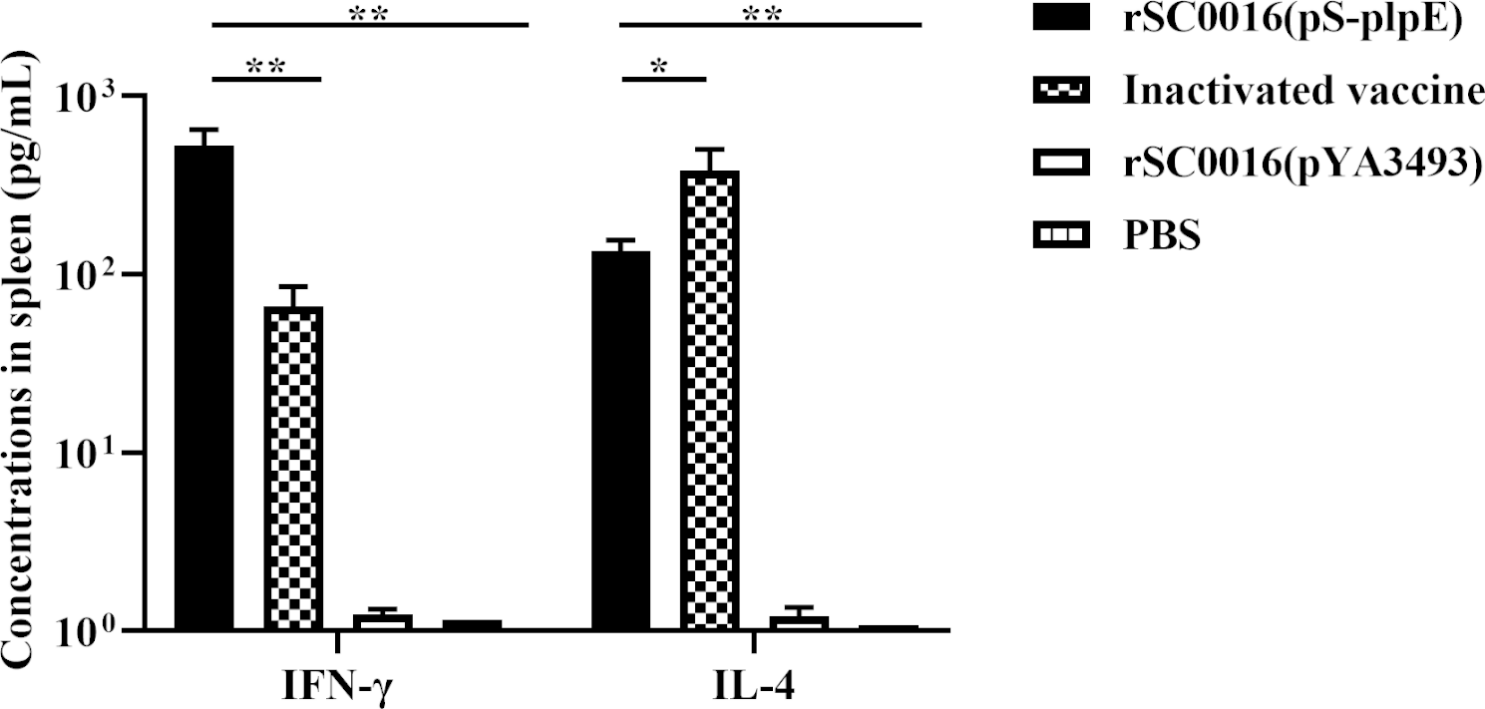
Levels of secreted IL-4 and IFN-γ were assayed by ELISA (n = 15). Splenic lymphocytes were used to evaluate cytokine secretion in vitro following re-stimulation with purified PlpE protein. The results are expressed as the mean ± SD. Degrees of significance are indicated as follows: *P < 0.05; **P < 0.01; n.s., nonsignificant (*P* ≥ 0.05)

Assessment of cell-mediated immune responses was done through measuring the IFN-γ and IL-4 cytokines by ELISA in culture supernatants fluid of splenic lymphocytes stimulated with PlpE (Fig. 4). The results revealed that the levels of IFN-γ and IL-4 in both the rSC0016(pS-PlpE) group and inactivated vaccine group were significantly higher compared with PBS and empty vector controls. These results show that both rSC0016(pS-PlpE) and inactivated vaccine immunization groups induced robust Th1 and Th2 type immune responses. A highly significant Th-1 response was observed, evident by the higher IFN-γ levels induced, in the rSC0016(pS-PlpE) group compared to the inactivated vaccine group’s response, while the inactivated vaccine group responded with a significant Th-2 response, shown by the higher IL-4 concentration, compared to the rSC0016(pS-PlpE) group, which would be expected for a parenterally administered antigen.

### Oral immunization with rSC0016(pS-PlpE) vaccine candidate strain increased survival and reduced pathological lung lesions in mice

**Fig. 5:**
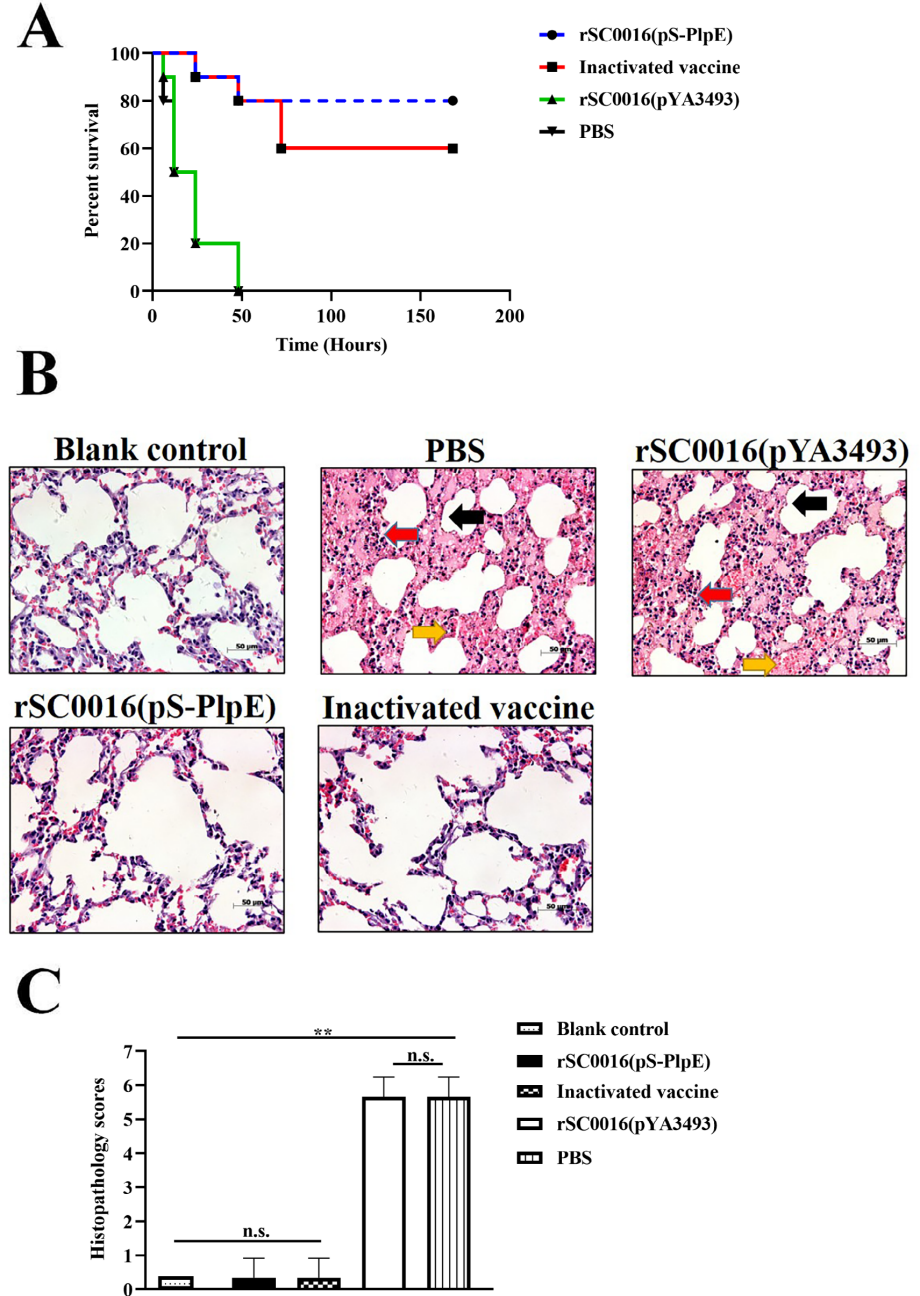
Protective efficacy of developed vaccine. (**A**) Survival rates of mice after *P. multocida* challenge were determined (n = 30). (**B**) Histological changes of lung tissue in mice (n = 15). HE staining revealed that mild inflammatory cell infiltration (red arrow), hyperemia (yellow arrow), and thickening of alveolar wall (black arrow) were observed in the lung tissues of PBS and rSC0016(pYA3493) immunized mice, compared with the blank control group. As expected, there were no obvious differences in the pathological changes among the rSC0016(pS-PlpE), the inactivated vaccine, and the blank control groups. (**C**) Histological scores of mice lungs in each group

Mice of each immunized group were intraperitoneally challenged with the isolated *P*. *multocida* serotype A three weeks after the second booster vaccination to verify the protective effect of the rSC0016(pS-PlpE) vaccine candidate strain. As shown in Fig. 5A, the results indicated that the survival rate was 80% in the rSC0016(pS-PlpE) group and 60% in the inactivated vaccine group. All mice in the control (empty vector-vaccinated and PBS-treated) groups died within 48 h after challenge. Pulmonary histopathologic changes upon *P*. *multocida* infection were assessed to further characterize the protective efficacy of the different vaccines compared to the PBS and empty vector control groups (Fig. 5B). Histological analysis of the lungs revealed that those in the rSC0016(pS-PlpE) and inactivated vaccine groups were similar to the non-infected blank control, with no obvious pathologic changes observed. In addition, the pathology scores for both the rSC0016(pS-PlpE) and inactivated vaccine groups were not significantly different from that of the non-infected blank control group (Fig. 5C). In contrast, compared with the blank control group, the lungs of mice in the PBS and empty vector groups challenged with the wild-type *P. multocida* strain displayed severe tissue injury, which mainly presented as mild inflammatory cell infiltration (red arrow), hyperemia (yellow arrow), and thickening of alveolar wall (black arrow) (Fig. 5B). Consistent with these histopathological changes, the lung lesion scores in the PBS and empty vector groups were significantly higher compared to those of the blank control group, rSC0016(pS-PlpE) group and inactivated vaccine group (Fig. 5C). These results suggest that rSC0016(pS-PlpE) vaccine may have protected mice against an i.p. challenge with wild-type *P*. *multocida* by preventing severe injury to the lungs.

## Discussion

*P. multocida* is an important zoonotic pathogen that has caused severe economic losses in the global swine industry and endangered public health around the world [28]. As with other opportunistic pathogens, *P*. *multocida* has the potential to proliferate and infect the lungs when host immunity is compromised due to stress or other pathogens infections [6, 7]. Because *P*. *multocida* can infect the host through mucosal tissues, the induction of protective immunity at mucosal tissues plays an important role in defense against pathogens [29]. At the same time, both cellular and humoral immune responses also play important roles in the host’s defense against *P*. *multocida* infections, which may be helpful to reduce the lungs damage and prompt their recovery [30]. In brief, the simultaneous induction of strong mucosal, humoral and cellular immune responses by vaccination against *P*. *multocida* colonization and infection is the goal for the development of a new vaccine.

Recently, numerous studies have shown that several *P*. *multocida* outer membrane proteins (OMPs) contribute to the pathogenicity and possess immunogenic and bactericidal properties [31, 32]. PlpE is an antigenic, surface-exposed and conserved OMP that is detected among the diverse serotypes of *P*. *multocida* investigated and has been mooted as a potential candidate for vaccine development [16, 33]. The use of attenuated *Salmonella* as a vector carrying heterologous antigen represents a novel strategy to preventing and controlling infectious diseases of humans and animals [24–27, 34]. In this study, a recombinant-attenuated *S.* Choleraesuis strain carrying the *plpE* gene inserted into the pYA3943 plasmid was constructed, and the expression and secretion of the PlpE protein from the rSC0016 strain carrying the *plpE* gene in the pYA3943 plasmid was demonstrated by WB analysis. The pYA3943 prokaryotic plasmid contains the pBR ori and *asd* genes, which produce a balanced lethal system to complement the chromosomal *asd* mutation strain. Our results show that the pS-PlpE plasmid can be stably maintained for over 50 passages in the rSC0016 vaccine candidate strain. These results to construct and characterize rSC0016(pS-PlpE) are consistent with results from previously reported literature [24, 27].

Besides expressing the target protein, the ability of the candidate vaccine strain to induce the antigen-specific antibody is equally important. In this study, mice orally inoculated with rSC0016(pS-PlpE) and administered the inactivated vaccine subcutaneously induced significantly higher levels of PlpE-specific IgG antibodies, while mice that received the empty vector, or PBS orally had no detectable specific antibodies. It is now widely accepted that clearance of pathogens is mediated by IgG that promote uptake and killing by professional phagocytes, mainly activated macrophages [35]. However, previous studies reported that the capsule of *P. multocida* is an important physical barrier that protects the bacteria to escape from the killing system of macrophages [36, 37]. Additionally, a previous study demonstrated that IgG levels against the PlpE protein are not indicative of protection, so the production of other antibody types should be further evaluated [13]. sIgA is the predominant immunoglobulin secreted at the mucosal surfaces and is the central aim of mucosal immune response [38]. Several studies have shown that sIgA can bind to pathogens and prevent them from attaching to or penetrating superficial mucosa layer, such as *Streptococcus pneumoniae*, *Neisseria meningitidis*, and *Bordetella pertussis* [39–41]. Moreover, enhanced mucosal immunity also resulted in higher protection against *P*. *multocida* [29, 42]. Our results revealed that after immunization, the level of sIgA measured from the rSC0016(pS-PlpE) group was slightly higher than levels in mice subcutaneously provided the inactivated vaccine, and was significantly higher than that of the empty vector or PBS groups. The increased level of sIgA in the rSC0016(pS-PlpE) group may be due to the method of immunizing by the oral route. Accumulating evidences suggests that oral vaccines can induce a more robust mucosal immunity than parenteral immunization [19]. However, the present study showed that the level of IgG, IgA, and IgG1 antibodies produced after subcutaneous immunization of mice with inactivated vaccine was comparable to the rSC0016(pS-PlpE) group, and the IgG2a level was significantly higher in the rSC0016(pS-PlpE) group at 5-weeks post oral vaccination. The significantly increased level of IgG2a for the rSC0016(pS-PlpE) group may be associated with the fact that the PlpE protein expressed by the recombinant vaccine is a very potent surface-exposed and secreted lipoprotein making it readily available to the host immune system. Previous studies have shown that lipoproteins serve as good mucosal adjuvants, stimulating high levels of IgG and secretory IgA production without additional adjuvants [43–46].

Cellular immune responses also play an important role in mitigating *P*. *multocida* infections [30]. IgG2a and IgG1 are used as indicators for Th1 and Th2 responses, respectively, thus the vaccine-induced T-cell phenotype can be evaluated by the levels of both IgG1 and IgG2a isotypes [47]. We observed the production of IgG1 and IgG2a isotypes in both rSC0016 (pS-PlpE) and inactivated vaccine immunized groups. These results suggest that both vaccines induced the Th1- and Th2-type immune responses against the PlpE antigen. Studies show that the generation of immune types may be related to the vaccination route and the formulation of the vaccine (live vs. inactivated) [48]. In previous studies, recombinant attenuated *Salmonella* vectors were more likely to induce Th1 immune types [24, 27]. IFN-γ and IL-2 are generally related to Th1-type cellular immune responses and can induce IgG2a production [49, 50]. IL-4 levels are generally related to Th2 type cellular immune responses and can stimulate IgG1 production [51, 52]. Similarly, both the rSC0016 (pS-PlpE) and inactivated vaccine immunization groups induced high levels of IFN-γ and IL-4 production. These results show that the rSC0016(pS-PlpE) recombinant vaccine induced potent, mixed Th1/Th2 cellular immune responses. In a previous study, the immunogenicity and protective effects of a *P. multocida* ghost vaccine was investigated, and the results showed that the ghost vaccine induced a higher level of cellular immune response compared to inactivated vaccines, which, in turn, protected mice against *P. multocida* infection, decreased tissue lesions and lowered bacterial loads [53]. Therefore, high levels of cellular immune response may also contribute to protection against *P. multocida* infection.

To assess whether the immune response induced by the vaccine candidate strain rSC0016 (pS-PlpE) was protective, mice were challenged with wild-type *P. multocida* by intraperitoneal injection three weeks post booster vaccination. Our data demonstrated that immunization with the rSC0016(pS-PlpE) vaccine twice provided 80% protection, compared with 60% protection provided by the inactivated vaccine. This is consistent with a previous study that parenteral inactivated vaccines only provide partial protection compared to live attenuated vaccines against *P. multocida* infection in mice [11]. In addition, compared with the inactivated vaccine group, the rSC0016(pS-PlpE) vaccine candidate strain also delayed the time to death in mice. This agrees with data from other studies, suggesting that formalin can destroy subtle antigenic structure on the outer-membrane of *P*. *multocida*, which leads to a lower immunogenic response [10, 18]. We further investigated pulmonary histopathology upon *P*. *multocida* infection for assessing the efficacy of rSC0016(pS-PlpE) to reduce lung tissue damage. The results showed that mice in the rSC0016(pS-PlpE) vaccine and the inactivated vaccine immunization groups had significantly reduced lung lesions and lung inflammation compared to the empty vector- and PBS-inoculated groups. These results were further validated by an evaluation of the pathology scores. In contrast to the blank control group, no significant changes of alveolar structural damage were observed in both the rSC0016(pS-PlpE) and inactivated vaccine groups. However, inflammatory cell infiltration, hyperemia, and alveolar wall thickening were observed in the PBS and empty vehicle immunization groups. Histopathological results showed that the rSC0016(pS-PlpE) vaccine candidate immunized group significantly reduced the pathological damage to the lungs of mice after challenge, which may be related to the high survival rate after *P*. *multocida* challenge.

## Conclusions

In summary, we constructed a novel live vector vaccine strain of *S.* Choleraesuis expressing PlpE of *P*. *multocida* and explored its potential application as a live vaccine in a mouse infection model. Oral inoculation of rSC0016(pS-PlpE) induced good induction of humoral immune responses, mixed Th1 and Th2 immune responses, and most importantly, mucosal immune responses in mice vaccinated twice with the live recombinant vaccine. Furthermore, immunized mice in the rSC0016(pS-PlpE) group exhibited significantly fewer lung lesions and 80% survival against lethal challenge with the wild-type *P*. *multocida* strain. These results suggest that attenuated *Salmonella* Choleraesuis may represent a valuable platform for the development of an oral vaccine for *P*. *multocida* infections, and for use as an effective strategy against multiple serotypes. Nonetheless, it is crucial to note that the outcomes observed in mice cannot be extrapolated to pigs [54, 55]. Hence, further studies are necessary to verify the protective efficacy of rSC0016(pS-PlpE) in pigs.

## Methods

### Mice and ethics statement

Female Balb/c mice were purchased from Yangzhou University Comparative Medicine Centre (Jiangsu, China). All animal experiments were performed in strict accordance with the animal welfare standards of the Animal Research Committee Guidelines of Jiangsu Province (License Number: SYXK (SU) 2017–0007), and approved by the Ethics Committee for Animal Experimentation of Yangzhou University. The mice were euthanized by CO_2_ method when necessary. For the animal experiments, all efforts were taken to minimize the suffering of the animals. All experiments with animals were performed in accordance with the ARRIVE guidelines.

### Bacterial strains, plasmids, and culture conditions

The strains and plasmids used in this work are listed in Table 1. *Escherichia coli* (*E*. *coli*) DH5α competent cells were used for subcloning experiments. Bacterial strains *E*. *coli* χ7213 and plasmids pYA3493 were kindly provided by R. Curtiss III (The Biodesign Institute, Arizona State University, Tempe, AZ). This plasmid encodes β-lactamase type II signals that, when fused in frame to an antigen gene of interest, directs antigen secretion to the periplasm and culture supernatant. *P*. *multocida* serotype A wild-type clinical strain separated in our laboratory were used as templates with full length primers of *plpE*-F and *plpE*-R (Table 2) to amplify *plpE* gene. Trypticase Soy Agar (TSA) or Trypicase Soy Broth (TSB) with 5% (v/v) fetal bovine serum was used for *P*. *multocida* strain cultivation. The *S*. Choleraesuis strain C78-3 (CVCC79103) was purchased from China Veterinary Culture Collection Center (CVCC, Beijing, China). *E*. *coli* and *S.* Choleraesuis strain C78-3 were cultured in Luria-Bertani (LB) liquid media or on LB agar (1.5% agar). In the case of rSC0016 strain, 2,6-diaminopimelic acid (DAP; 50 μg/mL) was added to the LB medium. When required, antibiotics and supplements were used at the following concentrations: Ampicillin (Amp; 50 μg/mL), Chloramphenicol (Cm; 50 μg/mL), Kanamycin (Kan; 50 μg/mL), D-mannose (0.2% wt/vol), L-arabinose (0.2% wt/vol) and D-lactose (1% wt/vol).

**Table 1:** Bacterial strains and plasmids used in this study

Strains and plasmid	Characteristics	Sources, references, or function
*E. coli* strains		
BL21	For expression the recombinant plasmids	Invitrogen
χ7213	*thi‑1 thr‑1 leuB6 fhuA21 lacY1 glnV44 asdA4 recA1 RP4 2‑Tc::Mu pir*	Provided by Dr. Roy Curtiss III
*S.* Choleraesuis		
C78-3	Wild type, virulent, CVCC79103	China Institute of Veterinary Drugs Control
rSC0016	ΔP_crp527_ :: TT *araC* P_BAD_*crp* Δ*pmi-2426* Δ*relA199*::*araC* P_BAD_*lacI* TT Δ*sopB1686* Δ*asdA33*	[27]
*Pasteurella multocida*		
*Pasteurella multocida* isolate	Wild-type, serotype A, field strain, virulent	Field isolate (stored in our lab), preparation of inactivated vaccine and challenge test in mice
Plasmids		
pYA3493	Plasmid Asd+; pBR *ori*, β-lactamase signal sequence-based periplasmic secretion plasmid	[22]
pET-28a	Expression vector, Kan^r^	Novagen
pMD19-T	Cloning vector; Amp^r^	TaKaRa
pET-28a-PlpE	A recombinant expression vector containing PlpE; Kan^r^	This study
pS-PlpE	pYA3493 with PlpE	This study

Kan^r^, Kanamycin resistance; Amp^r^, Ampicillin resistance

**Table 2:** The primers information

Primers	Sequences (5’–3’)	References
*plpE*-F	CCGGAATTCTGTAGCGGTGGTGGCGGTAGCGCTG	This study
*plpE*-R	ACGCGTCGACTTATTGTGCTTGGTGACTTTT	
pYA3493-F	AACGCTGGTGAAAGTAAAAGATG	This study
pYA3493-R pET-28a-F	CAGACCGCTTCTGCGTTCT	
	TAATACGACTCACTATAGGG	This study
pET-28a-R	GCTAGTTATTGCTCAGCGG	
*KMT*-F	ATCCGCTATTTACCCAGTGG	[57]
*KMT*-R	GCTGTAAACGAACTCGCCAC	
*hyaD*-*hyaC*-F	TGCCAAAATCGCAGTCAG	[57]
*hyaD*-*hyaC*-R	TTGCCATCATTGTCAGTG	

Underlined nucleotides denote enzyme restriction sites

### Bacterial identification, genotyping and virulence assay

*P*. *multocida* serotype A field strain was isolated from diseased pigs in Jiangsu Province, China. The genomic DNA of bacteria was extracted from overnight cultures of *P*. *multocida* isolate using the boiling method [56]. Serotype-specific primers or capsular typing primers were synthesized via referring to previous literature to identify *P*. *multocida* clinical strain [57]. The CFU of *P*. *multocida* field strain was calculated and then subjected to 10-fold dilution from 5.0 × 10^4^ to 5.6 × 10^0^ CFU. Six-week-old female BALB/c mice were randomly divided into 5 groups and mice (n = 5) in each group were challenged intraperitoneally (i.p.) with 0.1 mL of various dose of bacteria, and survival was recorded over a 14-day period. The 50% lethal doses (LD_50_) of the challenge strains were calculated according to the method of Reed and Muench [58].

### Protein expression, purification and polyclonal antibodies

The target gene *plpE* was subcloned into the pET-28a plasmid (Novagen) to generate the recombinant plasmid pET-28a-PlpE. The recombinant plasmid was then confirmed by restriction analysis using *Eco*R I and *Sal* I (Takara), PCR and sequencing by TsingKe Biological Technology. Next, BL21 (DE3) (Invitrogen) was transformed with pET-28a-PlpE, which was cultured at 37 °C until the OD_600_ reached 0.5, and IPTG (Sangon) was added to a final concentration of 0.5 mM to induce PlpE expression. The PlpE protein was affinity purified by Ni-affinity chromatography on Ni-NTA matrix as described previously [24]. Protein concentration was estimated using the BCA Protein Assay Kit (Thermofisher) and confirmed by Western Blotting (WB) with anti-His mAb (GenScript). Polyclonal antiserums of PlpE were prepared by injecting six-week-old female BALB/c mice intramuscularly with purified PlpE protein. Each injection contained 50 μg protein mixed 1:1 (V/V) with the Quick Antibody-Mouse 3 W adjuvant (Biodragon Immunotechnologies) as recommended by the producers and booster doses with the same formulated PlpE were given at two weeks later. After a week of the boost immunization, blood was collected through submandibular vein puncture for serum preparation and tested by iELISA.

### Construction of candidate vaccine strain and phenotyping properties

The *plpE* gene was amplified by PCR using primers that introduced *Eco*R I and *Sal* I restriction sites at the ends of the fragment. The resulting gene fragment was then inserted into the pYA3493 prokaryotic expression plasmid in the proper multiple cloning site to fuse PlpE with the β-lactamase type II secretion signal sequence, enabling PlpE expression. The success of the construction of pS-PlpE (pYA3493-PlpE) plasmid was verified by PCR, enzyme digestion, and sequencing. Upon confirmation, recombinant pS-PlpE plasmid and empty plasmid pYA3493 were introduced into rSC0016 strain by electroporation and Asd^+^ transformants were selected on LB plates. Expression of recombinant PlpE protein in the vaccine vector was verified by Western blotting. The plasmid stability of the rSC0016(pS-PlpE) was determined by a continuous passage experiment (one passage every 12 h) as described previously [27]. rSC0016(pYA3493) and C78-3 were used as control strains. Growth curves results were averaged across the three trials.

### Vaccine preparation

The vaccine strains used in this study were prepared as the method described in previous studies [24, 27]. Briefly, rSC0016(pS-PlpE), rSC0016(pYA3493) were grown in LB broth to a cell density of approximately 1 × 10^9^ CFU/mL (late log phase). The growth culture for the vaccine strains were supplemented with 0.2% arabinose and 0.2% mannose (Sigma). Bacterial cells were harvested by centrifugation at 7,000 × g for 10 min at 25 °C, and the pellet was resuspended in 300 μL of phosphate-buffered saline (PBS). The number of CFU were determined by plating serial dilutions onto solidified LB medium. Meanwhile, The *P*. *multocida* inactivated vaccine was prepared according to a published protocol as a control [53]. The *P*. *multocida* serotype A wild-type strain was incubated for 12 h at 37 °C in 5 mL TSB medium containing 5% (v/v) fetal bovine serum (Gibco). The revived culture was then added to same media as described above and incubated for 24 h at 37 °C. the bacterial suspension was then enumerated and the suspension adjusted, such that it contained 2 × 10^9^ CFU/mL. The bacterial suspension was inactivated by incubation with 0.4% formaldehyde (v/v) at 37 °C for 24 h. The inactivated bacterial pellet was mixed with an equal volume of oil/water adjuvant and emulsified by using emulsification mixer (Fluko, China) to generate the inactivated *P*. *multocida* vaccine. The sterility test was also qualified to ensure that the inactivated vaccine did not contain live bacteria.

### Vaccination and challenge procedure

The 195 female BALB/c mice at six-week-old were randomly divided into 5 groups. Group 1 (n = 45) and 2 (n = 45) were orally inoculated with 1 × 10^9^ CFU of rSC0016(pS-PlpE) or rSC0016(pYA3493), respectively, and group 3 (n = 45) was oral immunization with PBS as an infected control [59]. The Group 4 (n = 45) of mice were inoculated subcutaneously with 0.2 mL of *P*. *multocida* vaccine as a control vaccine [60]. Finally, Group 5 serves as a blank control (without any interventions, mainly for pathological histological analysis). Booster immunization was carried out three weeks after the primary immunization following the same vaccine formulation and methods as with primary inoculation. Three weeks after the boost immunization, the immunized BALB/c mice in different groups were infected by intraperitoneal injection with the virulent strain of *P*. *multocida* serotype A [60]. All animal experiments were repeated at least three times, and the data were pooled and analyzed together.

### Measuring antibody responses

To measure humoral and mucosal immune responses to vaccination, sera and vaginal lavages were collected seven days post-immunization (primary and boost immunization) for iELISA [24, 27]. To this end, 100 μL of purified PlpE proteins (1 ug/ml) as a capture molecule was coated in 96-well ELISA plates at 4 °C overnight. The ELISA plates were washed five times with PBST (350 μL/well) and blocked with 100 μL/well 5% skim milk for 2 h at room temperature. Test samples (serum or vaginal lavages) were twofold diluted in PBST (350 μL/well), added in duplicate, and incubated for 2 h at 37 °C. Following incubation, the plates were washed with PBST (350 μL/well), and HRP-conjugated goat anti-mouse IgG, IgG1, IgG2a, and IgA (Sigma, St. Louis, MO, USA) were added into the plates (100 μL/well) followed by 2 h incubation at 37 °C. The plates were visualized with 3,3′,5,5′-tetramethy1 benzidine (TMB) (Solarbio, Beijing, China) in the dark for 15 min at 37 °C, and the reaction was stopped with 2 M H_2_SO_4_. The antibody titre was expressed as the greatest serum dilution where OD_450_ was at least twice that of control sample at the same dilution.

### Cytokine assays

The spleens from each group of mice were collected one week after the boost immunization, and the spleen lymphocytes were isolated according to the instructions of the mouse spleen lymphocyte separation kit (Dakewe Biotech, China). Next, spleen lymphocytes were seeded into 96-well plates at a density of 5 × 10^5^ cells, and cultured with recombinant PlpE proteins (5 μg/mL) for 48 h. The concentration of IFN-γ and IL-4 in the culture supernatants was measured by Mouse IFN-γ and IL-4 ELISA kits (Solarbio, Beijing, China) following the manufacturer instructions. All assays were performed in triplicate.

### Pathological examination

Histopathological examination (n = 15) was performed as previously described [24, 27]. The lung tissues from the mice were fixed in 4% paraformaldehyde, and then dehydrated, embedded in paraffin and sectioned. Eventually, the sections were stained with Hematoxylin and Eosin (HE). The representative sections of lung were examined and scored to assess tissue pathology, as previously described [61]. In brief, histopathological scores were mainly based on interstitial inflammation, vascular endotheliitis, bronchitis, edema, serous effusion and thrombosis. All parameters were scored separately from 0 (absence of lesions) to 3 (severe lesions).

### Statistical analysis

Statistical comparisons among multiple groups were performed by one-way analysis variance (ANOVA). Analyses were conducted using Graph Pad Prism 8.0 software (Graph Pad Software Inc., CA, USA). A *P-*value of less than or equal to 0.05 (*) were considered significant difference, and a *P-*value of less than or equal to 0.01 (**) represents a highly significant difference. *P*-values larger than 0.05 were considered to be not significant (n.s.).

### Electronic supplementary material

Below is the link to the electronic supplementary material.

Supplementary Material 1

Supplementary Material 2

## Data Availability

The original PCR and whole blot pictures are available in Supplementary material (1) The DNA sequences used are listed in Supplementary material (2) All data generated or analyzed during this study are available from the corresponding author by request.

## Abbreviations

*P*. *multocida*: *Pasteurella multocida*
*S*. Choleraesuis: *Salmonella* Choleraesuis
CFU: Colony-forming units
HE: Hematoxylin and Eosin
i.p.: Intraperitoneally
iELISA: Indirect enzyme-linked immunosorbent assay
IPTG: Isopropyl-β-d-thiogalactoside
LB: Luria-Bertani broth
LD_50_: 50% lethal dose
Ni-NTA: Nickel-nitrilotriacetic acid
OMPs: Outer membrane proteins
PAR: Progressive atrophic rhinitis
PN: pneumonic pasteurellosis
PlpE: *Pasteurella* lipoprotein E
s.c.: Subcutaneously
Th1: Type 1 T helper
Th2: Type 2 T helper
TSA: Trypticase Soy Agar
TSB: Trypicase Soy Broth
WB: Western Blot

## References

[1] Wilson BA, Ho M (2013). *Pasteurella multocida*: from zoonosis to cellular microbiology. Clin Microbiol Rev.

[2] Harper M, Boyce JD, Adler B (2006). *Pasteurella multocida* pathogenesis: 125 years after Pasteur. FEMS Microbiol Lett.

[3] Carter GR (1955). Studies on *Pasteurella multocida*. I. A hemagglutination test for the identification of serological types. Am J Vet Res.

[4] Rimler RB, Rhoades KR (1987). Serogroup F, a new capsule serogroup of *Pasteurella multocida*. J Clin Microbiol.

[5] Peng Z, Wang H, Liang W, Chen Y, Tang X, Chen H, Wu B (2018). A capsule/lipopolysaccharide/MLST genotype D/L6/ST11 of *Pasteurella multocida* is likely to be strongly associated with swine respiratory disease in China. Arch Microbiol.

[6] Lung O, Ohene-Adjei S, Buchanan C, Joseph T, King R, Erickson A, Detmer S, Ambagala A (2017). Multiplex PCR and microarray for detection of Swine Respiratory Pathogens. Transbound Emerg Dis.

[7] Turni C, Meers J, Parke K, Singh R, Yee S, Templeton J, Mone NK, Blackall PJ, Barnes TS (2021). Pathogens associated with pleuritic pig lungs at an abattoir in Queensland Australia. Aust Vet J.

[8] Snyder E, Credille B (2020). *Mannheimia haemolytica* and *Pasteurella multocida* in bovine respiratory disease: how are they changing in response to efforts to control them?. Veterinary Clin North Am Food Anim Pract.

[9] Liu D, Chai T, Xia X, Gao Y, Cai Y, Li X, Miao Z, Sun L, Hao H, Roesler U (2012). Formation and transmission of *Staphylococcus aureus* (including MRSA) aerosols carrying antibiotic-resistant genes in a poultry farming environment. Sci Total Environ.

[10] Ahmad TA, Rammah SS, Sheweita SA, Haroun M, El-Sayed LH (2014). Development of immunization trials against *Pasteurella multocida*. Vaccine.

[11] Guan LJ, Song JJ, Xue Y, Ai X, Liu ZJ, Si LF, Li MY, Zhao ZQ. Immune Protective Efficacy of China’s Traditional Inactivated and Attenuated Vaccines against the Prevalent Strains of *Pasteurella multocida* in Mice. Vaccines 2021, 9(10). 10.3390/vaccines9101155 PMC8537324 34696263

[12] Dessalegn B, Bitew M, Asfaw D, Khojaly E, Ibrahim SM, Abayneh T, Gelaye E, Unger H, Wijewardana V (2021). Gamma-Irradiated Fowl Cholera Mucosal Vaccine: potential vaccine candidate for safe and effective immunization of Chicken against Fowl Cholera. Front Immunol.

[13] Mostaan S, Ghasemzadeh A, Asadi Karam MR, Ehsani P, Sardari S, Shokrgozar MA, Abolhassani M, Nikbakht Brujeni G (2021). *Pasteurella multocida* PlpE protein polytope as a potential subunit vaccine candidate. Vector borne and zoonotic diseases (Larchmont NY).

[14] Okay S, Ozcengiz E, Ozcengiz G (2012). Immune responses against chimeric DNA and protein vaccines composed of plpEN-OmpH and PlpEC-OmpH from *Pasteurella multocida* A:3 in mice. Acta Microbiol Immunol Hung.

[15] Zhang Y, Lin L, Yang J, Lv Q, Wang M, Wang F, Huang X, Hua L, Wang X, Chen H (2022). Two *Bordetella bronchiseptica* attenuated vaccine candidates confer protection against lethal challenge with *B. Bronchiseptica* and *Pasteurella multocida* toxin in mouse models. Vaccine.

[16] Hatfaludi T, Al-Hasani K, Gong L, Boyce JD, Ford M, Wilkie IW, Quinsey N, Dunstone MA, Hoke DE, Adler B (2012). Screening of 71 *P. multocida* proteins for protective efficacy in a fowl cholera infection model and characterization of the protective antigen PlpE. PLoS ONE.

[17] Wu JR, Shien JH, Shieh HK, Chen CF, Chang PC (2007). Protective immunity conferred by recombinant *Pasteurella multocida* lipoprotein E (PlpE). Vaccine.

[18] Okay S, Özcengiz E, Gürsel I, Özcengiz G (2012). Immunogenicity and protective efficacy of the recombinant *Pasteurella* lipoprotein E and outer membrane protein H from *Pasteurella multocida* A:3 in mice. Res Vet Sci.

[19] Gayet R, Bioley G, Rochereau N, Paul S, Corthésy B. Vaccination against *Salmonella* infection: the mucosal way. Microbiol Mol biology reviews: MMBR 2017, 81(3). 10.1128/MMBR.00007-17 PMC5584317 28615285

[20] Galen JE, Curtiss R (2014). The delicate balance in genetically engineering live vaccines. Vaccine.

[21] Curtiss R, Wanda SY, Gunn BM, Zhang X, Tinge SA, Ananthnarayan V, Mo H, Wang S, Kong W (2009). *Salmonella enterica* serovar typhimurium strains with regulated delayed attenuation in vivo. Infect Immun.

[22] Kang HY, Srinivasan J, Curtiss R (2002). Immune responses to recombinant pneumococcal PspA antigen delivered by live attenuated *Salmonella enterica* serovar typhimurium vaccine. Infect Immun.

[23] Wang S, Kong Q, Curtiss R (2013). New technologies in developing recombinant attenuated *Salmonella* vaccine vectors. Microb Pathog.

[24] Li Q, Lv Y, Li YA, Du Y, Guo W, Chu D, Wang X, Wang S, Shi H (2020). Live attenuated *Salmonella enterica* serovar Choleraesuis vector delivering a conserved surface protein enolase induces high and broad protection against *Streptococcus suis* serotypes 2, 7, and 9 in mice. Vaccine.

[25] Li Y, Wang S, Scarpellini G, Gunn B, Xin W, Wanda SY, Roland KL, Curtiss R (2009). Evaluation of new generation *Salmonella enterica* serovar typhimurium vaccines with regulated delayed attenuation to induce immune responses against PspA. Proc Natl Acad Sci USA.

[26] Li Y, Wang S, Xin W, Scarpellini G, Shi Z, Gunn B, Roland KL, Curtiss R (2008). A *sopB* deletion mutation enhances the immunogenicity and protective efficacy of a heterologous antigen delivered by live attenuated *Salmonella enterica* vaccines. Infect Immun.

[27] Li YA, Ji Z, Wang X, Wang S, Shi H (2017). *Salmonella enterica* serovar Choleraesuis vector delivering SaoA antigen confers protection against *Streptococcus suis* serotypes 2 and 7 in mice and pigs. Vet Res.

[28] Wilkie IW, Harper M, Boyce JD, Adler B. *Pasteurella multocida*: Diseases and Pathogenesis. In: *Pasteurella multocida: Molecular Biology, Toxins and Infection* edn. Edited by Aktories K, Orth JHC, Adler B. Berlin, Heidelberg: Springer Berlin Heidelberg; 2012: 1–22. 10.1007/82_2012_216 22643916

[29] Kang TL, Velappan RD, Kabir N, Mohamad J, Rashid NN, Ismail S (2019). The ABA392/pET30a protein of *Pasteurella multocida* provoked mucosal immunity against HS disease in a rat model. Microb Pathog.

[30] Doan TD, Wang HY, Ke GM, Cheng LT. N-terminus of Flagellin Fused to an Antigen improves vaccine efficacy against *Pasteurella multocida* infection in chickens. Vaccines 2020, 8(2). 10.3390/vaccines8020283 PMC7349934 32517250

[31] Luo Q, Kong L, Dong J, Zhang T, Wang H, Zhang R, Lu Q, Chen H, Shao H, Jin M (2019). Protection of chickens against fowl cholera by supernatant proteins of *Pasteurella multocida* cultured in an iron-restricted medium. Avian pathology: journal of the WVPA.

[32] Leeanan TEK, Pannoi R, Anuntasomboon S, Thongkamkoon P, Thamchaipenet P (2017). OmpA protein sequence-based typing and virulence-associated gene profiles of *Pasteurella multocida* isolates associated with bovine haemorrhagic septicaemia and porcine pneumonic pasteurellosis in Thailand. BMC Vet Res.

[33] Adler B, Bulach D, Chung J, Doughty S, Hunt M, Rajakumar K, Serrano M, van Zanden A, Zhang Y, Ruffolo C (1999). Candidate vaccine antigens and genes in *Pasteurella multocida*. J Biotechnol.

[34] Zhang XL, Jeza VT, Pan Q (2008). *Salmonella typhi*: from a human pathogen to a vaccine vector. Cell Mol Immunol.

[35] Takaya A, Yamamoto T, Tokoyoda K (2019). Humoral immunity vs. *Salmonella*. Front Immunol.

[36] Maheswaran SK, Thies ES (1979). Influence of encapsulation on phagocytosis of *Pasteurella multocida* by bovine neutrophils. Infect Immun.

[37] Rimler RB, Register KB, Magyar T, Ackermann MR (1995). Influence of chondroitinase on indirect hemagglutination titers and phagocytosis of *Pasteurella multocida* serogroups a, D and F. Vet Microbiol.

[38] Gutzeit C, Magri G, Cerutti A (2014). Intestinal IgA production and its role in host-microbe interaction. Immunol Rev.

[39] Hellwig SM, van Spriel AB, Schellekens JF, Mooi FR, van de Winkel JG (2001). Immunoglobulin A-mediated protection against *Bordetella pertussis* infection. Infect Immun.

[40] van der Pol W, Vidarsson G, Vilé HA, van de Winkel JG, Rodriguez ME (2000). Pneumococcal capsular polysaccharide-specific IgA triggers efficient neutrophil effector functions via FcalphaRI (CD89). J Infect Dis.

[41] Vidarsson G, van Der Pol WL, van Den Elsen JM, Vilé H, Jansen M, Duijs J, Morton HC, Boel E, Daha MR, Corthésy B (2001). Activity of human IgG and IgA subclasses in immune defense against Neisseria meningitidis serogroup B. J Immunol (Baltimore Md: 1950).

[42] Kharb S, Charan S (2011). Mucosal immunization provides better protection than subcutaneous immunization against *Pasteurella multocida* (B:2) in mice preimmunized with the outer membrane proteins. Vet Res Commun.

[43] Brown LE, Jackson DC (2005). Lipid-based self-adjuvanting vaccines. Curr Drug Deliv.

[44] Mao L, Liu C, Liu JY, Jin ZL, Jin Z, Xue RY, Feng R, Li GC, Deng Y, Cheng H (2022). Novel synthetic lipopeptides as potential mucosal Adjuvants enhanced SARS-CoV-2 rRBD-Induced Immune Response. Front Immunol.

[45] Moyle PM, Toth I (2008). Self-adjuvanting lipopeptide vaccines. Curr Med Chem.

[46] Nikam PS, Kingston JJ, Belagal Motatis AK (2021). Oral co-administration of bivalent protein r-BL with U-Omp19 elicits mucosal immune responses and reduces *S*. Typhimurium shedding in BALB/c mice. Immunol Lett.

[47] Stevens TL, Bossie A, Sanders VM, Fernandez-Botran R, Coffman RL, Mosmann TR, Vitetta ES (1988). Regulation of antibody isotype secretion by subsets of antigen-specific helper T cells. Nature.

[48] Zimmermann P, Curtis N. Factors that influence the Immune response to vaccination. Clin Microbiol Rev 2019, 32(2). 10.1128/CMR.00084-18 PMC6431125 30867162

[49] Constant SL, Bottomly K (1997). Induction of Th1 and Th2 CD4^+^ T cell responses: the alternative approaches. Annu Rev Immunol.

[50] Schroder K, Hertzog PJ, Ravasi T, Hume DA (2004). Interferon-gamma: an overview of signals, mechanisms and functions. J Leukoc Biol.

[51] Swain SL, Weinberg AD, English M, Huston G (1990). IL-4 directs the development of Th2-like helper effectors. J Immunol (Baltimore Md: 1950).

[52] Habiela M, Seago J, Perez-Martin E, Waters R, Windsor M, Salguero FJ, Wood J, Charleston B, Juleff N (2014). Laboratory animal models to study foot-and-mouth disease: a review with emphasis on natural and vaccine-induced immunity. J Gen Virol.

[53] Ran X, Meng XZ, Geng HL, Chang C, Chen X, Wen X, Ni H (2019). Generation of porcine *Pasteurella multocida* ghost vaccine and examination of its immunogenicity against virulent challenge in mice. Microb Pathog.

[54] Kennedy MJ, Yancey RJ, Sanchez MS, Rzepkowski RA, Kelly SM, Curtiss R (1999). Attenuation and immunogenicity of ∆*cya* ∆*crp* derivatives of *Salmonella choleraesuis* in Pigs. Infect Immun.

[55] Stabel TJ, Mayfield JE, Morfitt DC, Wannemuehler MJ (1993). Oral immunization of mice and swine with an attenuated *Salmonella* choleraesuis [delta *cya-12* delta(*crp*-*cdt*)*19*] mutant containing a recombinant plasmid. Infect Immun.

[56] Nnolim NE, Mpaka L, Okoh AI, Nwodo UU. Biochemical and molecular characterization of a Thermostable Alkaline Metallo-Keratinase from *Bacillus* sp. Nnolim-K1. Microorganisms 2020, 8(9). 10.3390/microorganisms8091304 PMC7565512 32867042

[57] Townsend KM, Boyce JD, Chung JY, Frost AJ, Adler B (2001). Genetic organization of *Pasteurella multocida* cap loci and development of a multiplex capsular PCR typing system. J Clin Microbiol.

[58] Reed LJ, Muench H (1938). A simple method of estimating 50% endpoints. Am J Hygiene.

[59] Zhou G, Tian Y, Tian J, Ma Q, Huang S, Li Q, Wang S, Shi H (2022). Oral immunization with attenuated *Salmonella* Choleraesuis expressing the P42 and P97 antigens protects mice against *Mycoplasma hyopneumoniae* challenge. Microbiol Spectr.

[60] Li Y, Xiao J, Chang YF, Zhang H, Teng Y, Lin W, Li H, Chen W, Zhang X, Xie Q (2022). Immunogenicity and protective efficacy of the recombinant Pasteurella multocida lipoproteins VacJ and PlpE, and outer membrane protein H from P. multocida A:1 in ducks. Front Immunol.

[61] Wu C, Qin X, Li P, Pan T, Ren W, Li N, Peng Y (2017). Transcriptomic analysis on responses of murine lungs to *Pasteurella multocida* infection. Front Cell Infect Microbiol.

