# Supplementary material for: Recombinant-attenuated Salmonella enterica serovar Choleraesuis vector expressing the PlpE protein of Pasteurella multocida protects mice from lethal challenge
Source: BMC Vet Res. 2023 Aug 19;19:128. doi: 10.1186/s12917-023-03679-0 (PMC10439597; doi:10.1186/s12917-023-03679-0)

Original PCR results

PCR results in manuscript

KMT gene

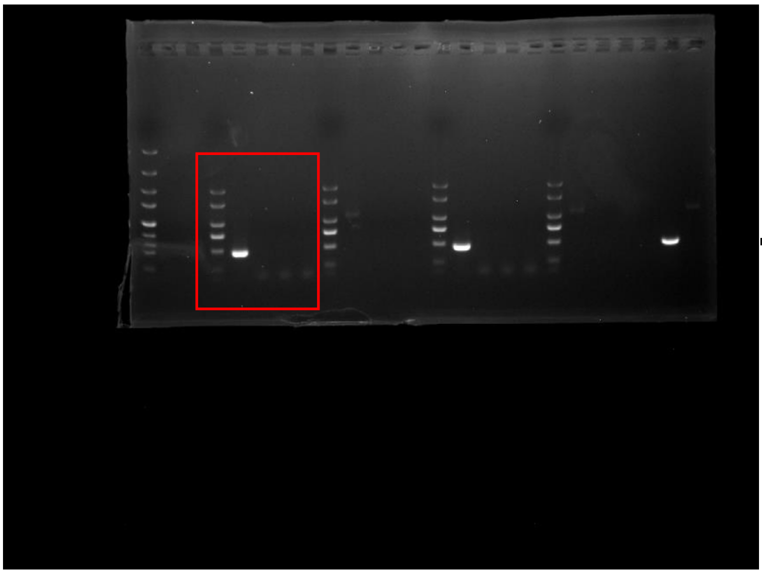

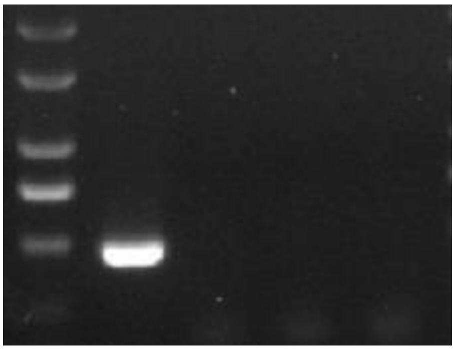

hyaD-*hyaC* gene

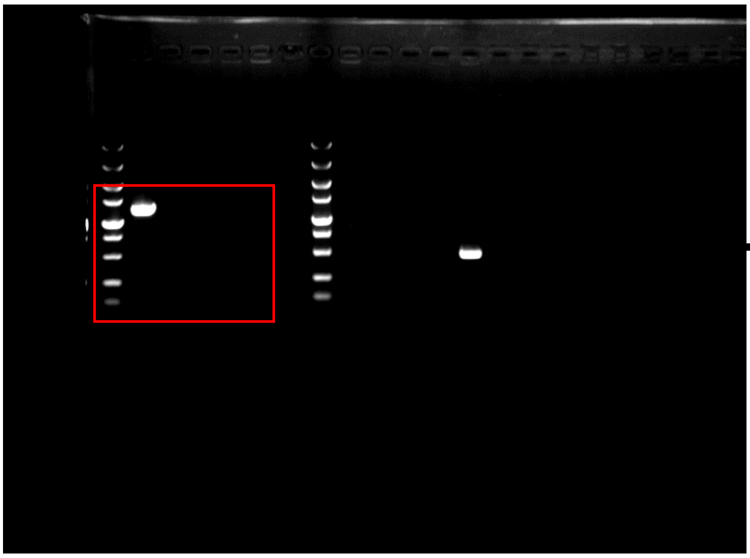

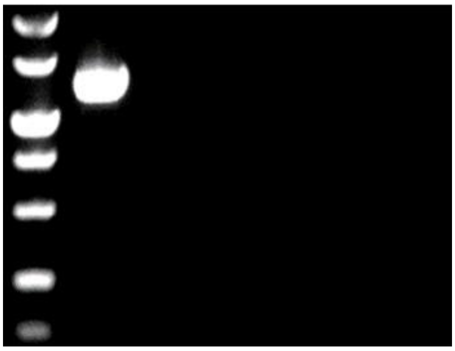

pS-PlpE PCR results

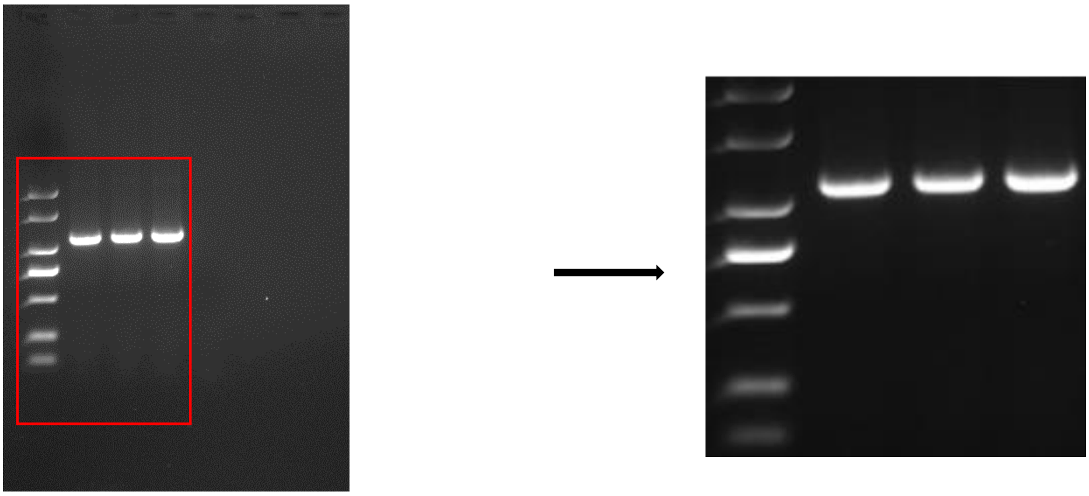

*Eco*R I and *Sal* I digested pS-PlpE

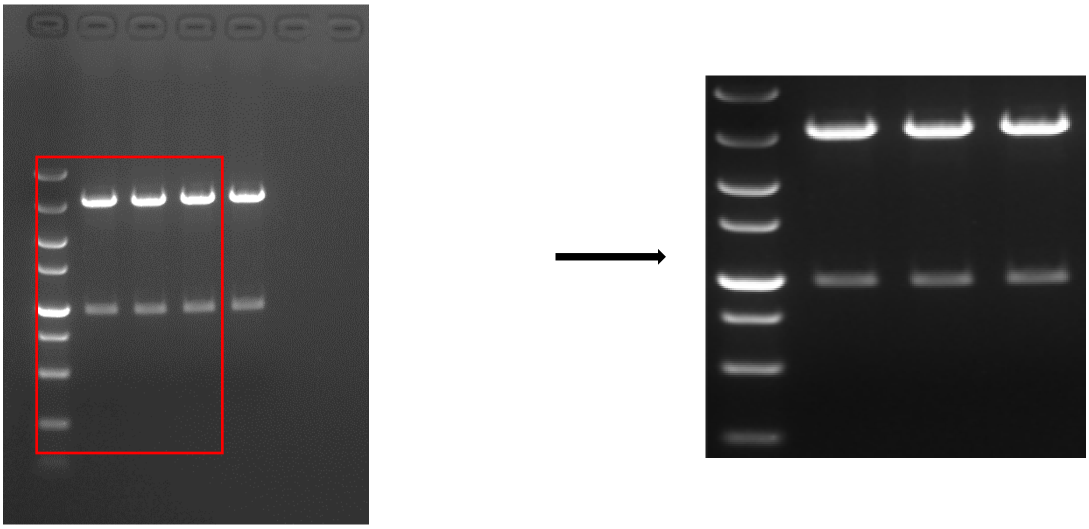

Plasmid stability of pS-PlpE during passage

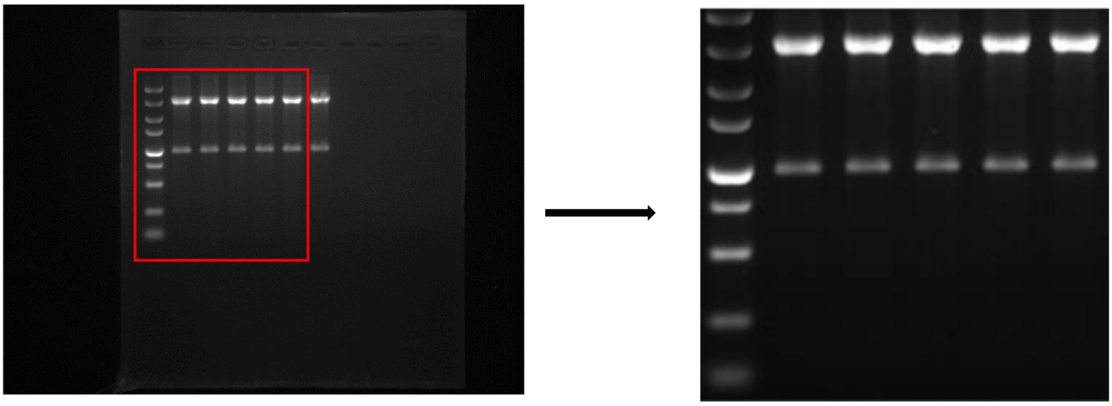

Original Western Blot

The expression of PlpE in rSC0016 was analyzed by WB

Western Blot in manuscript

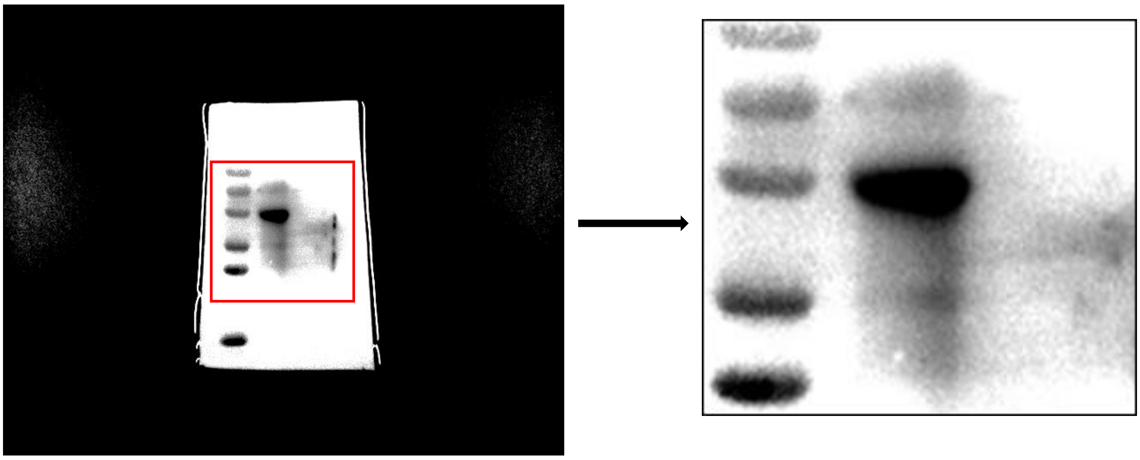

Supplement: Supplementary file 1 — Supplementary Material 1 [file 12917_2023_3679_MOESM1_ESM.pdf]
