# Supplementary material for: Recombinant-attenuated Salmonella enterica serovar Choleraesuis vector expressing the PlpE protein of Pasteurella multocida protects mice from lethal challenge
Source: BMC Vet Res. 2023 Aug 19;19:128. doi: 10.1186/s12917-023-03679-0 (PMC10439597; doi:10.1186/s12917-023-03679-0)
Supplement: Supplementary file 2 — Supplementary Material 2 [file 12917_2023_3679_MOESM2_ESM.pdf]

1. Identification results of strain species and serotypes

KMT gene:

ATCCGCTATTTACCCAGTGGGGCGGGGGGCATGACGATTGCCGCGAATTGAGTTTTATG
CCACTTGAAATGGGAAATGGCATTATTTTATGGCTCGTTGTGAGTGGGCTTGTCGGTAG
TCTTTTATTTGGCGTGTGGCAAAGAAAAGCACAGTTTTGTTGGGCGGAGTTTGGTGTG
TTGAGCCAATCTGCTTCCTTGACAACGGCGCAACTGATTGGACGTTATTTATTACTCAG
CTTATTGTTATTTGCCGGTTTATATTTCCTTGTCAGTCTGATTTATCAATATTTCCATGTTG
AGTTACGTTTCTTATGGCCATTATTGAAGCCATTAACGGCAGAGCGGTTTAATTTATTTAT
CGTGTATTGGTTACCTATTTTGGTCTTTTTCTTCGTGTTCAACGGTTTGATCGTGTCAGT
CCAAATGAAACAAAAAGTGGCGAGTCGTTTTTACAGC

hyaD-hyaC gene:

TGCCAAAATCGCAGTCAGTATTTTTTATCCCAATACATTAACGGCTTAGTGAAAAACTA
AACAATATTATTGAATATAATAAAAATATATTCGTTATTGTTCTACATGTTGATAAGAATC
ATCTTACACCAGATATCAAAAAAGAAATACTAGCCTTCTATCATAAACATCAAGTGAATA
TTTTACTAAATAATGATATCTCATATTACACGAGTAATAGATTAATAAAAACTGAGGCGC
ATTTAAGTAATATTAATAAATTAAGTCAGTTAAATCTAAATTGTGAATACATCATTTTTGA
TAATCATGACAGCCTATTCGTTAAAAATGACAGCTATGCTTATATGAAAAAATATGATGT
CGGCATGAATTTCTCAGCATTAACACATGATTGGATCGAGAAAATCAATGCGCATCCAC
CATTTAAAAAGCTCATTAAAACTTATTTTAATGACAATGACTTAAAAAGTATGAATGTGA
AAGGGGCATCACAAGGTATGTTTATGACGTATGCGCTAGCGCATGAGCTTCTGACGATT
ATTAAAGAAGTCATCACATCCTGCCAGTCAATTGATAGTGTGCCAGAATATAACACTGA
GGATATTTGGTTCCAATTTGCACTTTTAATCTTAGAAAAGAAAACCGGCCATGTATTTAA
TAAAACATCGACCCTGACTTATATGCCTTGGGAACGAAAATTACAATGGACAAATGAAC
AAATTGAAAGTGCAAAAAGAGGAGAAAATATACCTGTTAACAAGTTCATTATTAATAGT
ATAACTCTATAAAACACTTGCATTTTATTAAAAATAAAATCCTATAATATTTGCAGTTTAA
ATAAAGGATAAAAAATGAAGAAAATTACAATTGCTGGGGCTGGCTATGTTGGTTTATCC
AATGCAGTATTATTAGCTCAACACCACAATGTGATCTTATTAGATATTGATCAAAATAAA
GTTGATTTAATTAATAATAAAAAATCGCCCATCACAGATAAAGAAATCGAAGATTTCTTA
CAAAATAAATCACTGACAATGATGGCAA

2. The construction of recombinant plasmids (pS-PlpE and pET-2a-PlpE)

plpE gene:

TGTAGCGGTGGTGGCGGTAGCGCTGGAAATCGTGCTGACCGTGTAGAGGAAAAAGCA
CAACCGGTTCAATCAAATAGTGAGCCTTCTTCCACTCCAATCAAACATCCTATGACTAA
TAGTGCTACGAATACTTCTCTTCATGACAAACTTTCAATGTCTTCTCATGACACATCCAA
AGAAAATAGTCAACAATCCTCCTTTCAAGCCCCTCTAGAACAAGAAAAAAACCAACCT
GCACAAGAAAATCTTACTTGGACAGGTTATCATGTTTCAGAATGGGGAAATGCGAGTA
ATAATGTAGATAAAGATAATGTTACGGTATTCACTTTCGTAAAATATAATTCTCAATATAA
TGATGATCCAGTTTTTGATAAAACAAAAACACAAAGTAAAACGATATCATTAGTTGACG
GAAAAAATGAAAATAAAGAGCATTATTATCACTTTACGCTAAAAGACGATTTATTTTATT
ATGGCAGTTATGGACAACCTTCATCAGATTATAAAAAAATAGAAGAAAACTATATTTATG
CAATCAAACCAGATGCAATAAATAATGAGAACATCAATGCACTAACTGCAACTTACCAT
CAAGAAGATGGTTTTATATATTCCGTATTAAGTGATGTAAATCGAGTTGGTTCAGAATAT
ATTCCTCAGTATGGCAATGTGAGTCTTACTATACAAAATGGTAAAATTTATGGTGAGATT
TATAGGCATAACCGAGGGTACGATGATCTATTTAAGCTCTCTGGAGAAGGTCGGAATTT

AATATTAACACCACATAAAAATAACCCTTATGATCTTTCCCCTACAGGACCCGACAACAT
GACAATGGAGCTGAATTTTATCAACGCAGAAAAGACTGATAAAAAATACGTTGTTGGT
GTAGGAAAAGCTGAAAAATATTATGGGTTATTATTTGCTGAAAAAAGTCACCAAGCAC
AATAA
